# Patent challenges and factors associated with successful patent challengers under the patent linkage system: recent evidence from South Korea after the Korea United States free trade agreement

**DOI:** 10.1186/s12992-021-00765-6

**Published:** 2021-09-28

**Authors:** Kyung-Bok Son, Nahye Choi, Boram Lee, Joonsoo Byun, Dong-Wook Yang, Tae-Jin Lee

**Affiliations:** 1 Sungkyunkwan University, Seobu-ro, Jangan-gu, Suwon-si, Gyeonggi-do South Korea; 2 Department of Public Health Science, Graduate School of Public Health, Seoul National University, 1 Gwanak-ro, Gwanak-gu, 08826 Seoul, South Korea; 3 Institute of Health and Environment, Seoul National University, 1 Gwanak-ro, Gwanak-gu, 08826 Seoul, South Korea

**Keywords:** Patent linkage, Patent challenge, Prospecting, Free Trade Agreements, South Korea

## Abstract

**Objectives:**

The patent linkage system upgraded patent challenges to an important factor in granting timely market approval for generic drugs. We aim to understand patent challenges and identify the factors that are associated with successful patent challengers under the patent linkage system in South Korea.

**Methods:**

We constructed a novel dataset that combined information on manufacturers with detailed data about their patent challenges after introduction of the patent linkage system. Based on the number of successful patent challenges, manufacturers were categorized into non-challengers, passive challengers, and aggressive challengers. Then, two types of logistic models were applied to identify the factors associated with successful and aggressive challengers.

**Findings:**

Only 39 active ingredients were challenged by 77 manufacturers from March 2015 to December 2019. Of 171 manufacturers, 94 (55 %) were non-challengers, 58 (34 %) were passive challengers who had succeeded in fewer than 4 patent challenges, and 19 (11 %) were aggressive challengers who had succeeded in 4 or more patent challenges. Higher sales, more employees, and a greater number of reimbursed drugs were associated with being a patent challenger, while a greater number of reimbursed drugs was associated with being an aggressive challenger.

**Conclusion:**

Some manufacturers utilize patent challenges to strengthen their product portfolios in the market. However, under the patent linkage system, the frequency of patent challenges is limited in South Korea compared to the United States. In particular, patent challenges against drugs in injection form and biologics are very rare.

## Background

Patent challenges reflect conflict and competition [1]. Changes in the regulatory processes for generic drugs have made patent challenges more critical in the pharmaceutical sector [2]. Since the Hatch-Waxman Act, generic manufacturers in the United States can submit dossiers related to market approval for generics to the Food and Drug Administration (FDA) prior to expiration of the patent for the corresponding brand-name drug [3]. These generic manufacturers can assert that one or more brand-name drug patents are invalid and/or not infringed by their generic drugs. This type challenge is referred to as a “Paragraph IV challenge” [4].

Paragraph IV challenges have caused substantial changes in the behavior of the pharmaceutical industry [4]. Brand-name manufacturers have increased their acquisition of additional patents in order to delay generic entrants, in a process called “evergreening” [5]. Many of these additional patents are deemed invalid and/or lacking in applicability [6]. The courts have become less likely to conclude that additional patents are valid and/or infringed by generic manufacturers [6]. Patent challenges initiated by generic manufacturers for selectively targeted drugs with large sales volumes have been observed in the United States. Researchers describe this marketing strategy employed by generic drug manufacturers as “prospecting” [7, 8]. The debates about “evergreening” of brand-name manufacturers and “prospecting” of generic manufacturers have received a great deal of policy attention in the United States and have been empirically investigated by many researchers [4, 6–10].

**Fig. 1:**
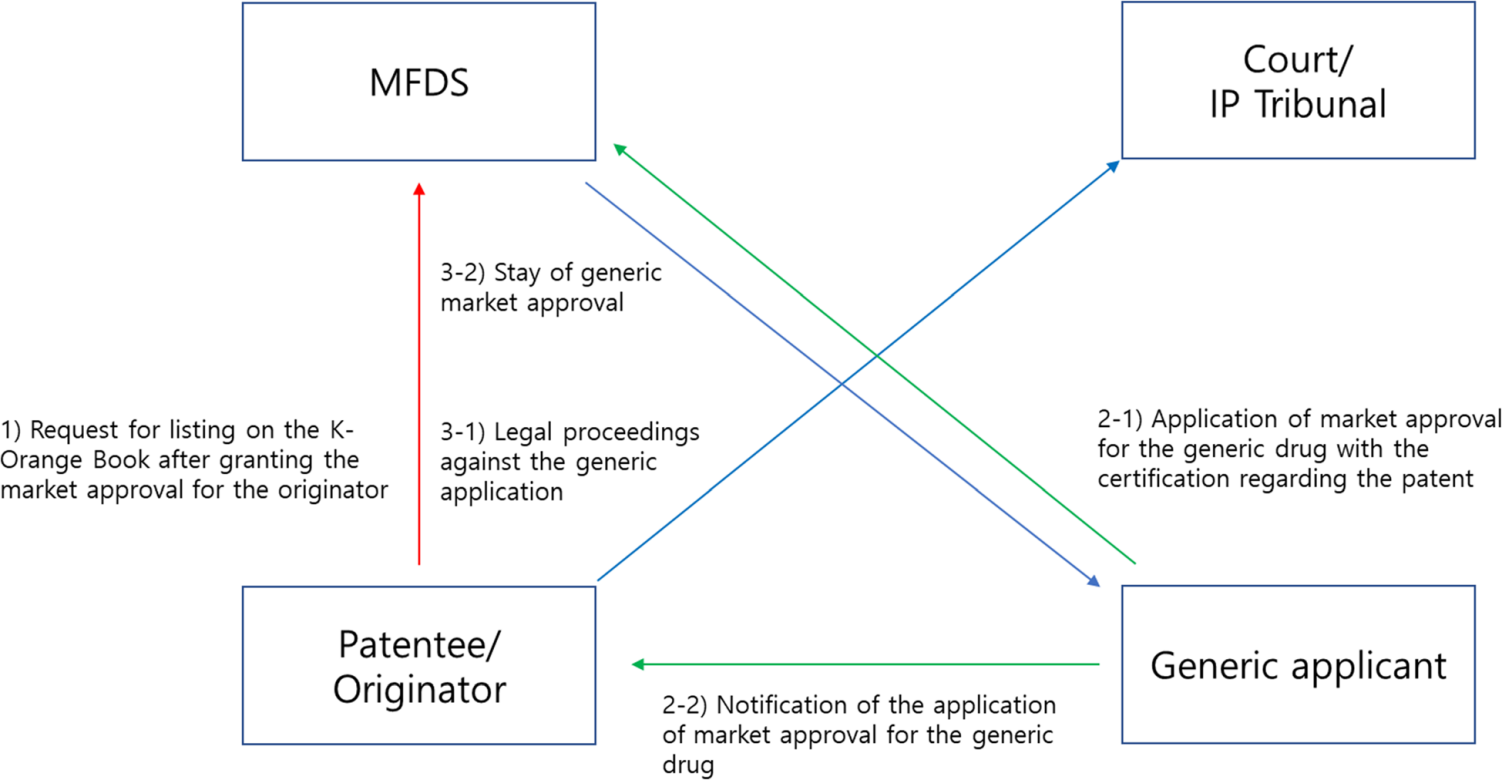
Schematic illustration of the South Korean patent linkage system. IP Tribunal: Intellectual Property Tribunal. MFDS: Ministry of Food and Drug Safety

South Korea introduced the patent linkage system in 2012 after signing the Korea United States Free Trade Agreement (KORUS) [11]. The system indicates a conditional relationship between the patent status of the originator drug and the granting market approval for a corresponding generic drug [12]. Figure 1 illustrates the patent linkage system in South Korea. Like the system in the United States, it consists of four parts [2]: (1) the patent list (the so-called “K-Orange Book”), (2) the notification process, which functions similar to “Paragraph IV challenges” in the United States, (3) stay of generic market approval, and (4) 9-month exclusivity for the first generic entrant. The originator submits information on a patent to be listed in the “K-Orange Book” to the Ministry of Food and Drug Safety (MFDS) within 30 days of approval of the originator drug. Only patents listed in the “K-Orange Book” are the subjects of the patent linkage system. A generic manufacturer must provide certification to the MFDS and notification to the marketing holder of the corresponding originator drug contending that the patent is invalid or will not be infringed. The notification may cause an action for infringement and then trigger a stay of generic market approval for up to 9 months unless litigation is concluded in the generic manufacturer’s favor. Meanwhile, 9-month exclusivity is provided for the first generic entrant, which is the first entrant for which a manufacturer submitted the related dossiers to the MFDS and successfully challenged related patents (or obtained a favorable decision from a court). This 9-month exclusivity period was established to provide an incentive for generic manufacturers to challenge the validity of patents.

Many researchers and civic activists have asserted that the patent linkage system would cause “evergreening” of brand-name drugs and delay generic entrance, leading to an increase in the price of originators due to their monopoly in the market [13–15]. Given the characteristics of the pharmaceutical market in South Korea, it was also reasonable to anticipate that the system might have a detrimental effect on the local pharmaceutical industry. Approximately 400 domestic manufacturers exist in the market, most of which produce generic drugs; the majority of these manufacturers are small- or medium-sized companies in terms of financial resources [16, 17]. In contrast, only a few domestic manufacturers have the capacity to develop new medicines [17]. The government wanted to secure time to introduce the new system and provide additional time for domestic manufacturers to respond to the system [18]. The system was introduced in two steps. First, in 2012, the government introduced (1) the patent list and (2) the notification process. Three years later, the government implemented the rest of the system: (3) stay of generic market approval; and (4) exclusivity for the first generic entrant.

In this study, we investigated the characteristics of drug manufacturers to understand patent challenges. Since 2012, the patent list, named the K-Orange Book, has been publicly accessible and easily searchable in South Korea. We aim to understand patent challenges and the factors associated with successful patent challengers under the patent linkage system. To this end, we examined all patent challenges initiated by domestic manufacturers against patents listed in the K-Orange Book, counted the number of successful patent challenges by each manufacturer from March 2015 to December 2019, and applied logistic regression to elucidate the determinants of patent challenges at the manufacturer level. More specifically, we sought to evaluate how frequently patent challenges occurred, what types of patents and/or brand-name drugs were challenged, and which manufacturers successfully introduced generic drugs into the market after successful patent challenges.

## Methods

### Subjects

We constructed a novel dataset that combined information on manufacturers with detailed data about their patent challenges after introduction of the patent linkage system. The unit of analysis was a domestic manufacturer. We began by identifying the complete list of domestic manufacturers that had been granted market approval for at least one drug from the MFDS website. Foreign manufacturers that had mainly introduced brand-name drugs to the South Korean market were excluded. At this stage, we identified 171 domestic manufacturers. Then, we counted each manufacturer’s successful patent challenges from March 2015 to December 2019. We also identified the characteristics of manufacturers using various resources. The number of new medicines, modified new medicines, and reimbursed drugs under the National Health Insurance (NHI) were obtained for each manufacturer from the MFDS and Health Insurance Review and Assessment Service (HIRA) database. The financial resources of the manufacturers, including sales and employees, were retrieved from the KIS-VALUE database of the National Information and Credit Evaluation (NICE). The names of all companies that have been designated innovative pharmaceutical manufacturers were obtained from the website of the Ministry of Health and Welfare.

### Study design

First, we retrieved information on active ingredients against patent challenges and the corresponding patent challengers to identify the major characteristics of active ingredients that have been challenged by generic manufacturers. Active ingredients against patent challenges were described by the following details: route of administration, anatomical therapeutic chemical (ATC) classification, reimbursement status, approved year, and market size. Market size was defined as sales of drugs in 2017 at the market grouped according to the third category of the ATC classification of the active ingredient [19].

Second, we identified the factors associated with being a successful patent challenger under the patent linkage system. The number of successful patent challenges per manufacturer was counted. Manufacturers were first split into two groups: manufacturers with a successful patent challenge and those without a successful patent challenge. A successful patent challenge was defined as the granting of market approval for a generic drug with a 9-month exclusivity period. Note that 9-month exclusivity is granted for the first manufacturer who has challenged a patent and received a favorable decision in court. Furthermore, we subdivided successful patent challengers into two groups based on the observed number of successful challenges: aggressive challengers and passive challengers. Aggressive challengers are manufacturers who have frequently utilized patent challenges for their business strategy under the patent linkage system. More specifically, aggressive challengers were defined as manufacturers who had succeeded in four or more patent challenges, while passive challengers were defined as manufacturers with less than four successful patent challenges.

### Model specification

Several factors determine whether a company begins a patent challenge [1, 6, 7, 9, 20, 21], including the value of the patent right, the cost of a trial, and the expectations for winning the trial. This study investigated the characteristics of manufacturers to determine the factors associated with being a successful patent challenger under the patent linkage system. There has been a lack of empirical investigation into the determinants of patent challenges at the manufacturer level. Thus, we chose a set of explanatory variables based on the relevant literature and formed a hypothesis.

First, we captured some important characteristics of manufacturers: their total sales, number of employees, and number of reimbursed drugs under the NHI. Sales and employees were used to represent the size of the manufacturers based on their financial and human resources. The number of reimbursed drugs under the NHI was used to present the manufacturers’ business strategies. Manufacturers with large numbers of reimbursed drugs were assumed to have adopted a product diversification strategy to maximize their profits in the market, while manufacturers with a small number of such drugs were assumed to have adopted a market penetration strategy with limited numbers of products to maximize their profits. Second, we constructed a variable representing the “innovative manufacturer” designation as a proxy for the firms’ research activities. As of 2019, the government designated 46 research-based manufacturers as innovative manufacturers and provided comprehensive support for these companies [22]. These manufacturers were assumed to have research capability. Similarly, we added a variable representing the experience of marketing new or modified new drugs as a proxy for development capability. In South Korea, a new drug is defined as “a drug composed of new materials, the chemical structure or the construction of substance of which is wholly unique, or a drug of composite medication containing new materials as effective ingredients” [23]. A modified new drug is “a drug that is not classified as a new drug but requires safety and efficacy review” [23]. For instance, drugs containing new salts as active substances and drugs with new indications, new compositions of active substances, new routes of administration, and new dosages are defined as modified new drugs [23]. Manufacturers that have marketed new or modified new drugs were assumed to have development capability.

### Empirical strategy

We began with descriptive analysis. We described all active ingredients that were challenged by generic manufacturers and categorized these active ingredients into three groups based on the number of challengers. Next, we presented the manufacturers and the number of successful patent challenges made by each one. Finally, we analyzed differences in the distribution of variables between manufacturers with and without a successful patent challenge using the Chi-squared test and t-test. We also sub-divided patent challengers into aggressive and passive challengers and presented the difference in the distribution of variables between these two sub-groups.

For the main analysis, we adopted two types of logistic models. The first logistic regression model used “being a successful challenger” as the outcome (Model I). We then selected patent challengers and applied the second logistic regression model, in which “being an aggressive challenger” was the outcome (Model II). P_*i*_ indicates the probability of the existence of at least one successful patent challenge between March 2015 and December 2019 for manufacturer *i* in model (I) Similarly, P_*i*_ indicates the probability that manufacturer *i* is an aggressive challenger in model (II) Five variables were added to the model: innovative, new drug, sales, employees, and reimbursed drugs. Table 1 describes the variables included in this study. Sales, employees, and reimbursed drugs were used after a log transformation. Data management and analysis were performed using R statistical software (version 3.4.3). Statistical significance was considered when p-values were less than 0.05.

**Table 1:** Description of variables

Variable	Description
Successful patent challenger	= 1 if a generic manufacturer, from 2015 to 2019, granted a marketing approval with 9-month exclusivity after challenging a patent and obtaining favorable decision at a court
Aggressive patent challenger	= 1 if a generic manufacturer, from 2015 to 2019, achieved 4 and more successful patent challenges
Innovative	= 1 if a generic manufacturer designated as innovative pharmaceutical manufacturers by the ministry of health as of 2019
New drug	= 1 if a generic manufacturer granted a marketing approval for new drug or modified new drug as of 2019
Sales	Total sales of a manufacturer in 2019 (million Korean Won)
Employees	Total employees of a manufacturer in 2019 (full time employees)
Reimbursed drugs	Total number of reimbursed drugs under the National Health Insurance that a manufacturer granted marketing approval as of 2019

$$\mathrm{Model}:\;\mathrm{Logit}\;\left({\mathrm P}_{\mathrm i}\right)\;=\;{\mathrm\beta}_0+\;{\mathrm\beta}_1\;{\mathrm{Innovative}}_{\mathrm i}\;+\;{\mathrm\beta}_2\;\mathrm{New}\;{\mathrm{Drug}}_{\mathrm i}\;\;+\;{\mathrm\beta}_3\;{\mathrm{Sales}}_{\mathrm i}\;+\;{\mathrm\beta}_4\;{\mathrm{Employees}}_{\mathrm i}+\;{\mathrm\beta}_5\;\mathrm{Reimbursed}\;{\mathrm{Drugs}}_{\mathrm i}+\;\in_{\mathrm i}$$

## Results

### Challenge of active ingredients by generic manufacturers

**Fig. 2:**
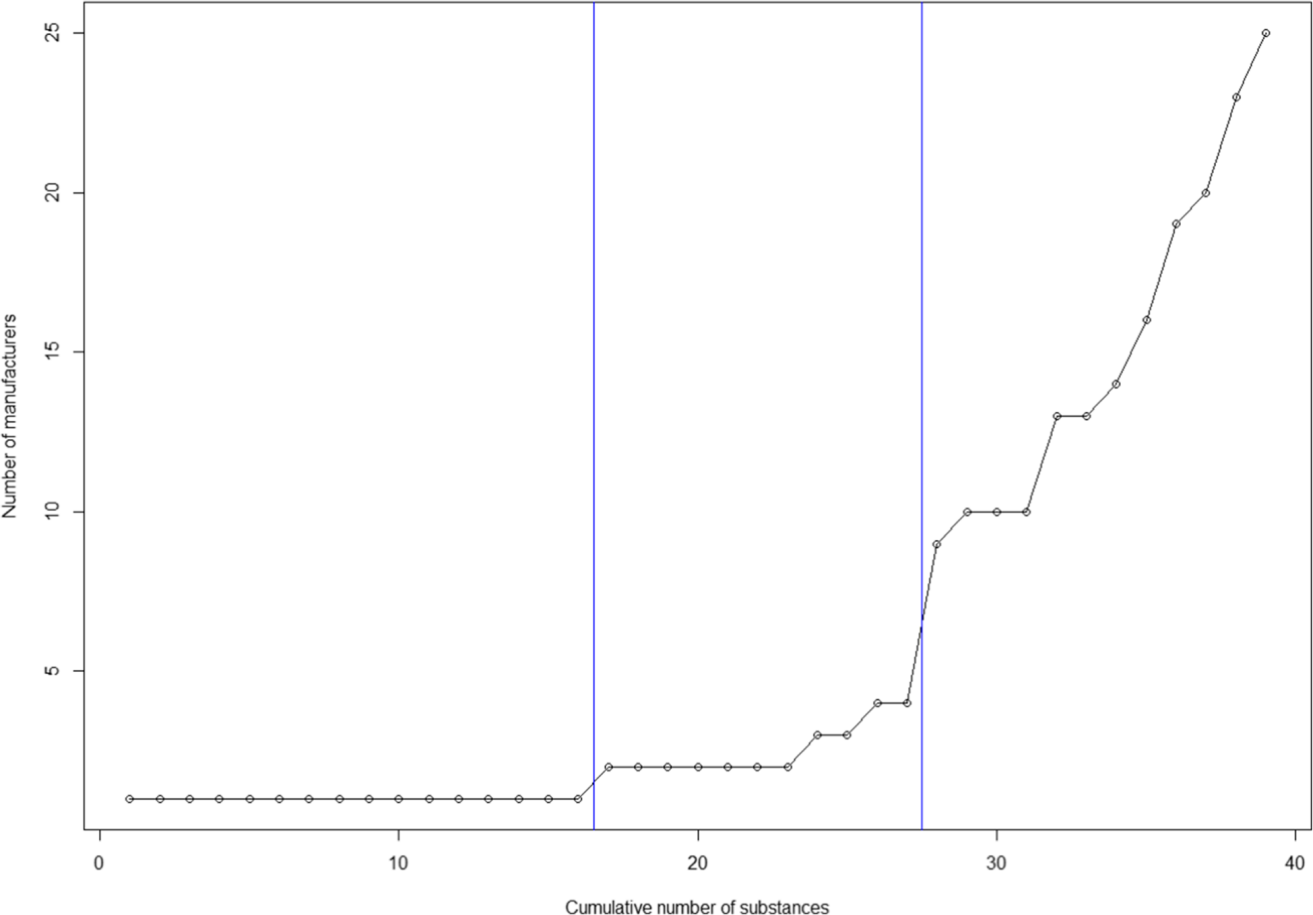
Cumulative numbers of substances and successful patent challengers

Figure 2 presents the cumulative number of active ingredients challenged by generic manufacturers and the number of successful patent challengers from March 2015 to December 2019. Of 39 active ingredients, 16 (41 %) were challenged by a sole manufacturer, 11 (28 %) were challenged by more than one but fewer than five manufacturers, and 12 (31 %) were challenged by more than eight manufacturers.

Table 2 describes the characteristics of active ingredients of brand name drugs according to the number of patent challengers. Of the 39 active ingredients of brand name drugs that were challenged, 32 (82 %) were in oral form, 18 (46 %) were classified as A/B/C according to the first category of ATC classification, 29 (74 %) were reimbursed under the NHI, and 18 (46 %) were approved between 2011 and 2016. Interestingly, none of these active ingredients was a biologic. Of the 16 active ingredients challenged by a single manufacturer, 9 (56 %) were in oral form, 15 (94 %) were reimbursed under the NHI, and 7 (44 %) were approved between 2011 and 2016. Conversely, 12 active ingredients were challenged by more than eight manufacturers. Of these 12 ingredients, all (100 %) were in oral form, 8 (75 %) were A/B/C according to the first category of ATC classification, 7 (58 %) were reimbursed under the NHI, and 7 (58 %) were approved between 2011 and 2016. Finally, 11 active ingredients were challenged by more than one but fewer than five manufacturers. Of these 11 ingredients, all (100 %) were in oral form, 7 (64 %) were reimbursed under the NHI, and 6 (55 %) were A/B/C according to the first category of ATC classification. Notably, all drugs in injection form and other non-oral forms were challenged by a single manufacturer. The proportion of active ingredients that accounted for sales of more than 700,000 million Korean won (KRW) increased as the number of challengers increased, from 18 % (3 out of 16) for one challenger to 54 % (6 out of 11) for fewer than five challengers and 58 % (7 out of 12) for more than eight challengers (Cochrane-Armitage test for trends, *p* = 0.029).

**Table 2:** Characteristics of substances subject to patent challenges

	Substances	Challenged by one manufacture		Challenged by less than 5 manufacturers		Challenged by more than 8 manufacturers	
	39	16	(41 %)	11	(28 %)	12	(31 %)
Route							
Oral	32	9	(28 %)	11	(34 %)	12	(38 %)
Injection	3	3	(100 %)	0	(0 %)	0	(0 %)
Others	4	4	(100 %)	0	(0 %)	0	(0 %)
ATC classification							
A/B/C	18	4	(22 %)	6	(33 %)	8	(44 %)
J/L	6	3	(50 %)	2	(33 %)	1	(17 %)
M/N	4	1	(25 %)	1	(25 %)	2	(50 %)
Others	11	8	(73 %)	2	(18 %)	1	(9 %)
Reimbursed							
No	10	1	(10 %)	4	(40 %)	5	(50 %)
Yes	29	15	(52 %)	7	(24 %)	7	(24 %)
Approved year							
1994–2005	8	4	(50 %)	4	(50 %)	0	(0 %)
2006–2010	13	5	(38 %)	3	(23 %)	5	(38 %)
2011–2016	18	7	(39 %)	4	(22 %)	7	(39 %)
Market size (million KRW)							
< 100,000	9	5	(56 %)	1	(11 %)	3	(33 %)
100,000-700,000	14	8	(57 %)	4	(29 %)	2	(14 %)
700,000 ≤	16	3	(19 %)	6	(38 %)	7	(44 %)

*ATC* Anatomical Therapeutic Chemical; *KRW* Korean won

### Manufacturers as patent challengers

**Fig. 3:**
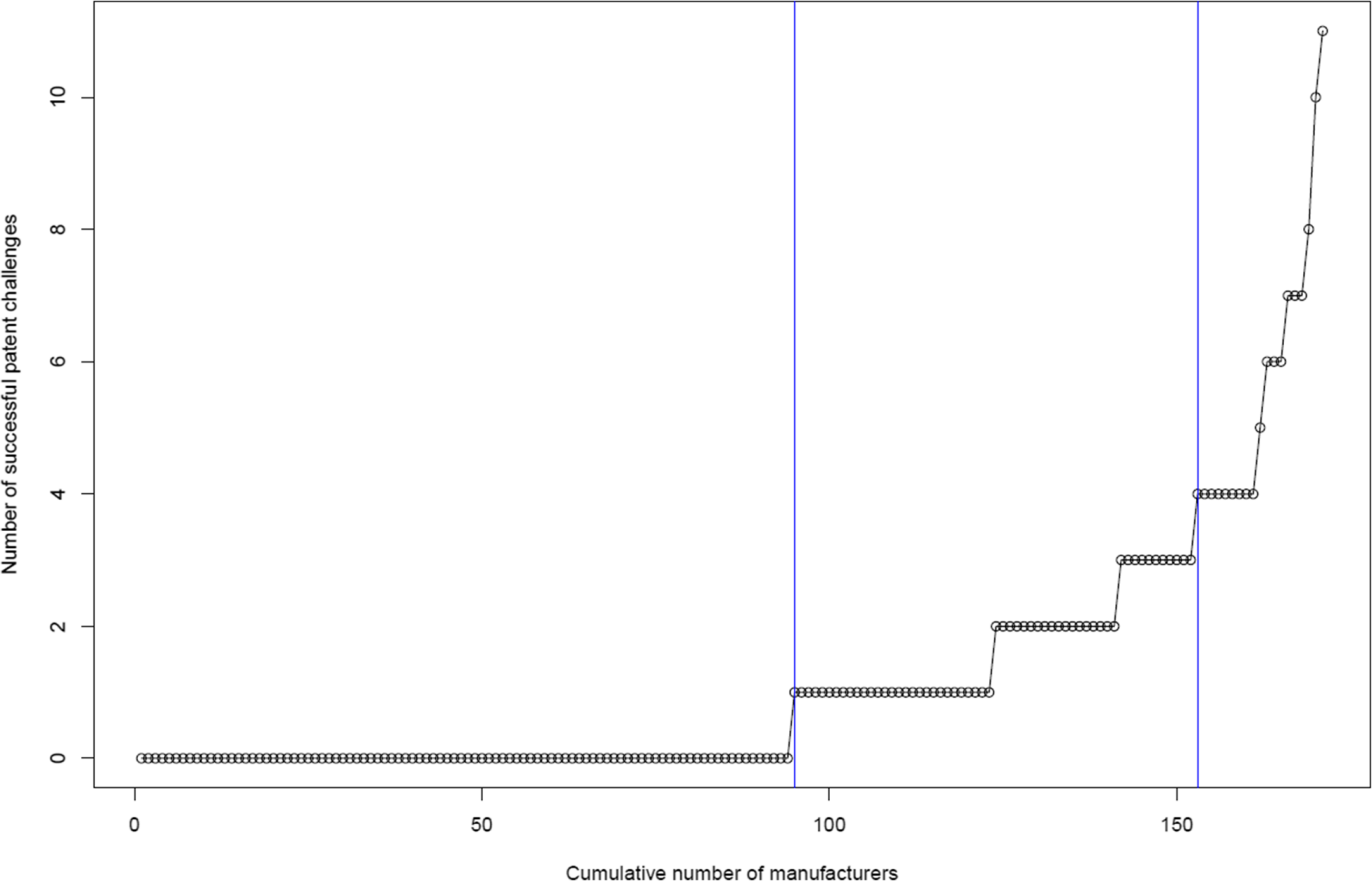
Cumulative numbers of manufacturers and successful patent challenges

Figure 3 presents the cumulative numbers of manufacturers and successful patent challenges. Of 171 manufacturers, 94 (55 %) were non-challengers, 58 (34 %) were passive challengers who had succeeded in less than 4 patent challenges, and 19 (11 %) were aggressive challengers who had succeeded in 4 or more patent challenges. Interestingly, 6 (3.5 %) had succeeded in 7 or more patent challenges.

Table 3 summarizes the characteristics of all manufacturers. First, we categorized the manufacturers into challengers and non-challengers. Of 77 challengers, 20 (31 %) were innovative manufacturers and 33 (43 %) had experience in marketing new or modified new drugs. Note that the variables “innovative” and “experience in developing new or modified new drugs” indicate the manufacturers’ research and development capability, respectively. Of 94 non-challengers, 9 (10 %) were innovative manufacturers and 9 (10 %) had experience in marketing new or modified new drugs. We found that challengers reported greater sales, employees, and numbers of reimbursed drugs than non-challengers. Next, we grouped challengers into aggressive and passive challengers. Of the 58 passive challengers, 12 (21 %) were innovative manufacturers while 22 (38 %) had experience in marketing new or modified new drugs. In contrast, out of 19 aggressive challengers, 8 (42 %) were innovative manufacturers while 11 (58 %) had experience in marketing new or modified new drugs. The variables innovative, new drugs, and sales did not significantly differ between aggressive and passive challengers. However, the variables employees and numbers of reimbursed drugs were different between the two groups. Aggressive challengers had more employees and greater numbers of reimbursed drugs than passive challengers.

**Table 3:** Characteristics of manufacturers

	Non challengers	Challengers	*p*-value	Passive challengers	Aggressive challengers	*p*-value
	94	77		58	19	
Innovative						
No	85	57	0.0083	46	11	0.1221
Yes	9	20		12	8	
New drug						
No	85	44	< 0.0001	36	8	0.2080
Yes	9	33		22	11	
Sales	87,321 (175,878)	213,598 (271,251)	0.0005	183,181 (263,621)	306,449 (280,190)	0.0856
Employees	239 (339)	515 (510)	< 0.0001	433 (436)	765 (637)	0.0448
Reimbursed drugs	73 (76.7)	189 (69.4)	< 0.0001	170 (56.9)	248 (72.3)	< 0.0001

### Regression analyses

Table 4 presents the results of the logistic models. We began by exploring the determinants of being a successful patent challenger. Model I showed that the odds of being a successful patent challenger increased with increased sales [AOR: 4.21, *p* = 0.0043] and increased number of reimbursed drugs [AOR: 6.84, *p* < 0.0001]. However, manufacturers with large numbers of employees had decreased odds of being a successful challenger [AOR: 0.27, *p* = 0.0153]. In addition, we found no evidence that research and development capabilities were associated with being a successful patent challenger. We next selected patent challengers and applied the second logistic model, in which the outcome was being an aggressive challenger. Model II showed that the odds of being an aggressive challenger increased with an increase in the number of reimbursed drugs [AOR: 31.75, *p* = 0.0048]. However, the variables of sales and employees failed to show significant associations with the outcome.

**Table 4:** Results of the logistic regression models

	Model I: Being a successful challenger *N* = 171			Model II: Being an aggressive challenger *N* = 77		
	AOR	95 % CI	P	AOR	95 % CI	P
Innovative						
Yes (Ref_no)	1.87	[0.42–9.42]	0.4159	0.90	[0.15–5.15]	0.9052
New drug						
Yes (Ref_no)	1.87	[0.62–5.98]	0.2705	0.89	[0.14–5.16]	0.9002
Sales	4.21	[1.60-11.81]	0.0043	1.57	[0.36–7.12]	0.5434
Employees	0.27	[0.09–0.77]	0.0153	1.00	[0.19–5.70]	0.9924
Reimbursed drugs	6.84	[3.29–17.66]	< 0.0001	31.75	[3.49-463.07]	0.0048

## Discussion

The patent linkage system requires early resolution of patent disputes before marketing approval for a generic drug is granted. South Korea introduced the patent linkage system in March 2012 after signing the KORUS. South Korea also devised a 9-month exclusivity to provide an incentive for generic manufacturers to challenge the validity of patents. In a nutshell, the patent linkage system upgraded patent challenges to an important factor in timely market approval of a generic drug. It is well-documented that patent challenges cause substantial changes in the behaviors of pharmaceutical manufacturers in the United States [6–10]. However, there has been little empirical evidence regarding the effect of the patent linkage system on the behaviors of manufacturers in South Korea. This was the first study to construct a novel dataset binding domestic manufacturers to their successful patent challenges and to analyze the determinants of being a patent challenger and/or an aggressive patent challenger at the manufacturer level. The findings of this study shed light on the behavior of pharmaceutical manufacturers in the era of the patent linkage system.

### Overall patent challenges

From March 2015 to December 2019, 39 active ingredients were challenged by 77 domestic manufacturers. Some active ingredients were challenged by one manufacturer, while others were challenged by more than eight manufacturers. The active ingredients that were the subjects of patent challenges varied in terms of route of administration, ATC classification, and reimbursement status under NHI. We were able to draw some interesting findings. First, market size was closely associated with the number of patent challengers according to the Cochrane-Armitage test for trends. This finding partially supports the notion that “prospecting” is common among generic manufacturers in South Korea, as it implies that challengers file in the hopes of entering a large market rather than a small market. In a similar vein, it was recently reported that the market size of the drug was associated with the number of generic entrants in the South Korean market [24]. Given these findings, we concluded that generic drug manufacturers are more likely to enter a large market than a small market [17]. Under the patent linkage system, challenging the validity of patents is a necessary step to obtain market approval of a generic drug prior to patent expiry. Next, patent challenges against drugs in injection and other forms were rare compared to those against drugs in oral form. Only 7 (18 %) out of 39 active ingredients were injectable drugs or drugs in other non-oral forms, and each of these ingredients was challenged by a single manufacturer. In South Korea, the route of administration of a drug was associated with the number of generic entrants [24]. Drugs in injection and other forms are often not candidates for patent challenge and/or development of generic entrants because of the complexity of the associated patents and/or production processes [24]. In a similar vein, none of the active ingredients was a biologic.

In 2011, the United States Congress instituted “inter partes review” to complement its slow and expensive patent litigation process [25]. The new system completes the process within one year of initiation and reduces the costs that are incurred to approximately 10 % of those incurred in the patent litigation process [26]. Note that the probability of patent challenges increases as the cost of a trial and/or the term of settlement in a court decreases [20, 21]. Researchers in the United States have systematically evaluated the use of “inter partes review” for patent challenges [25]. From September 2012 to April 2017, 362 reviews were initiated against 198 patents, which were associated with 134 new drug applications [25]. In a similar vein, other researchers have described a wide range of patent challenges initiated by generic manufacturers as “prospecting” in the United States [7, 8]. Interestingly, the frequency of patent challenges is lower in South Korea under the patent linkage system than in the United States. We found that 39 active ingredients were challenged by 77 generic drug manufacturers in South Korea during the study period, implying that, on average, 8 active ingredients were challenged by 16 generic manufacturers annually. A previous study also demonstrated that the frequency of patent challenges was relatively low in South Korea [18]. In contrast, 77 challenges against 42 patents associated with 29 new drug applications were observed per year in the United States [25].

### Determinants of patent challenges at the manufacturer level

Using logistic regression models, we elucidated the determinants of a successful patent challenger or an aggressive challenger at the manufacturer level. We used the variables of innovation and new drugs as proxies for manufacturer research and development capability, respectively. However, these two variables were not significantly associated with being a patent challenger. These observations implied that either patent challenges were not difficult for domestic manufacturers without research and development capability to initiate under the South Korean legal system, or manufacturers with research and development capability mainly concentrated on the development of new drugs rather than generics. Evidence supports the former hypothesis. First, we found that the majority of aggressive challengers were manufacturers with research and development capability. Next, small manufacturers can jointly initiate patent challenges with other manufacturers under the current patent linkage system. Finally, South Korea has a two-tier litigation system with both judicial courts and the Intellectual Property Tribunal [27]. Judicial courts have jurisdiction in cases of damages and injunctions against patent infringement, while the Intellectual Property Tribunal had jurisdiction over challenges to the validity of patents [28]. Reportedly, trials to challenge the validity of patents in the Intellectual Property Tribunal are not expensive and such trials can be completed in a timely manner [28].

Manufacturers with large sales volumes were more likely to be successful challengers than those with small sales volumes, implying that the former had more financial resources at their disposal for patent challenges. In a similar vein, manufacturers with a large number of reimbursed drugs under the NHI were more likely to be successful challengers than those with a small number of reimbursed drugs. Manufacturers with large numbers of reimbursed drugs might adopt a product diversification strategy to maximize their profits, while manufacturers with small numbers would be more likely to adopt a market penetration strategy with limited numbers of products to maximize their profits. More specifically, manufacturers with large numbers of reimbursed drugs might adjust their business strategies to maximize the chances of their products entering the market. Patent challenges provide an option to increase the diversity of their product portfolios. In a similar vein, manufacturers with large numbers of reimbursed drugs were more likely to be aggressive patent challengers. Finally, manufacturers with small numbers of employees were more likely to be patent challengers. These companies might perform joint patent challenges with other manufacturers. Thus, it is realistic to assume that manufacturers with limited numbers of employees might prefer outsourcing and/or co-patent challenges to filing their own sole patent challenges. It is also well-documented that the majority of generic manufacturers in South Korea utilize co-bioequivalence tests and/or outsource bioequivalence tests to other manufacturers as part of the process of introducing generics [29].

### Limitations

This study has some limitations. First, we analyzed the determinants of patent challenges at the manufacturer level. However, these determinants include the value of the patent right, the cost of a trial (and/or settlement) in a court, and the expectations of winning the trial. These factors were not controlled in this study. Since the introduction of the patent linkage system in 2012, the patent list, named the K-Orange Book, has been publicly accessible and easily searchable in South Korea. Given this circumstance, the value of the patent right, the cost of trial (and/or settlement), and the expectations of winning the trial were not quite different among generic manufacturers. Thus, this study focused on the characteristics of manufacturers in an attempt to understand patent challenges since the introduction of the system. Second, this study dealt with successful patent challenges, while failed patent challenges were excluded from the analysis. It may be necessary to include failed challenges to fully understand patent challenges in South Korea. However, many manufacturers initiated patent challenges regardless of their expectations of winning the trial during the initial stage of the system. In a similar vein, this study analyzed patent challenges that occurred from 2015 to 2019. Thus, the results presented in this study reflect only the short-term impacts of the patent linkage system. Finally, the patent linkage system has caused “evergreening” among brand-name drug manufacturers and “prospecting” among generic manufacturers. These two behaviors are closely associated with each other. For instance, an increase in the number of patents for brand-name drugs would cause an increase in patent challenges. However, this study did not consider the effect of “evergreening” of brand-name drugs on “prospecting” of generic drugs. Further research on “evergreening” of brand-name drugs is needed to fully evaluate the effect of the patent linkage system.

## Conclusions

The patent linkage system upgraded patent challenges to an important factor in timely market approval of a generic drug. Some manufacturers have utilized patent challenges to strengthen their product portfolios in the market. However, the frequency of patent challenges is limited in South Korea under the patent linkage system compared to the United States. In particular, patent challenges against drugs in injection form and biologics were very rare.

## Data Availability

The datasets used for the current study are available from the corresponding author on reasonable request.
